# Efficacy of DNA Intercalator‐Conjugated Triplex‐Forming Oligonucleotide as Anticancer Agent

**DOI:** 10.1002/cmdc.202500325

**Published:** 2025-09-14

**Authors:** Haruki Toyama, Akira Toriba, Atsushi Shibata, Takehiko Wada, Asako Yamayoshi, Yu Mikame

**Affiliations:** ^1^ Graduate School of Biomedical Sciences Nagasaki University 1‐14 Bunkyo‐machi Nagasaki Nagasaki 852‐8521 Japan; ^2^ Division of Molecular Oncological Pharmacy Faculty of Pharmacy Keio University 1‐5‐30 Shibakoen Minato‐ku Tokyo 105‐8512 Japan; ^3^ IMRAM (Institute of Multidisciplinary Research for Advanced Materials) Tohoku University 2‐1‐1 Katahira Aoba‐ku Sendai Miyagi 980‐8577 Japan; ^4^ Department of Life Science and Technology Institute of Science Tokyo 4259 Nagatsutacho Midori‐ku Yokohama Kanagawa 226‐8501 Japan

**Keywords:** anticancer, deoxyribonucleic acid, human epidermal growth factor receptor type2, intercalator, triplex‐forming oligonucleotide

## Abstract

A triplex‐forming oligonucleotide (TFO) can form a sequence‐specific triple helix via Hoogsteen hydrogen bonding to polypurine tracts within a major groove side of a DNA duplex. Triplex formation can induce a double‐strand break, and this phenomenon at the amplified gene loci can selectively induce the cell death of cancer cells with specific gene amplification. However, the relationship between the binding affinity of TFO for target gene loci and the cell death response remains unclear. In this study, it is aimed to develop DNA intercalator‐conjugated TFOs with higher affinity for the human epidermal growth factor receptor type2 gene, which is often amplified in breast cancer cells, than the unmodified TFO. The binding affinity of the TFOs for the target DNA duplex is analyzed using nondenaturing polyacrylamide gel electrophoresis, and one of the DNA intercalator‐conjugated TFOs show a higher binding affinity for the target duplex than the unmodified TFO. The cell death responses induced by these TFOs using the WST‐8 assay is also evaluated suggesting that the higher binding affinity of the TFO for amplified gene loci can lead to a stronger cell death response of cancer cells with specific gene amplification.

## Graphical abstract

The binding affinity of triplex‐forming oligonucleotide (TFO) is successfully improved for human epidermal growth factor receptor type2 (HER2) gene sequence by using DNA intercalator. The higher binding affinity of TFO for HER2 gene sequence are correlated with the anticancer activity induced by the TFO and resulted in stronger cell death response of HER2‐positive breast cancer cells.

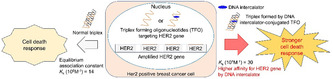

## 1 Introduction

The triplex‐forming oligonucleotide (TFO) can form a sequence‐specific triple helix via Hoogsteen hydrogen bonding to polypurine tracts within a major groove side of the DNA duplex.^[^ ^1^ ^]^ Triplex formation can be used to regulate gene functions by disturbing the interactions of DNA with protein complexes such as DNA polymerase and transcription factors.^[^ ^2^ ^]^ Other biological applications using TFO were also reported.^[^ ^3^ ^]^ Rogers et al. reported that triplex formation causes DNA perturbation, which interferes with replication fork progression, resulting in fork collapse and DNA double‐strand breaks. The efficiency of DSB induction by TFO is low because the endogenous DNA repairing system competes with this perturbation to avoid a DSB.^[^ ^4^ ^]^ However, the same research group demonstrated that targeting the amplified human epidermal growth factor receptor type2 (HER2) gene loci of HER2 positive breast cancer cells by TFO was very effective in inducing cell apoptosis,^[^ ^5^ ^]^ demonstrating the potential of TFO as a new drug platform for cancer therapy.

**Figure 1:**
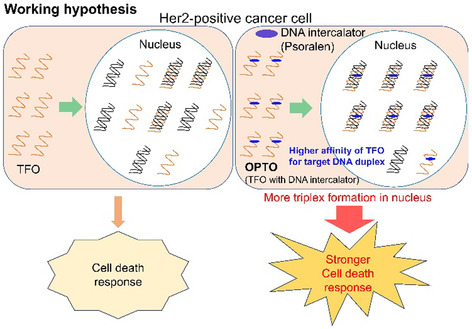
Working hypothesis of this work. The higher binding affinity of OPTOs for amplified gene loci will result in the formation of a larger number of triplexes in the nucleus, which will induce a stronger cell death response of cancer cells.

Our group decided to pursue this phenomenon because the relationships between the binding affinity of TFOs for target gene sequences and cell apoptosis response were not mentioned, and we hypothesized that a higher binding affinity of the TFO for HER2 gene loci can induce a stronger cell death response of HER2 positive breast cancer cells (**Figure** **1**).

**Figure 2:**
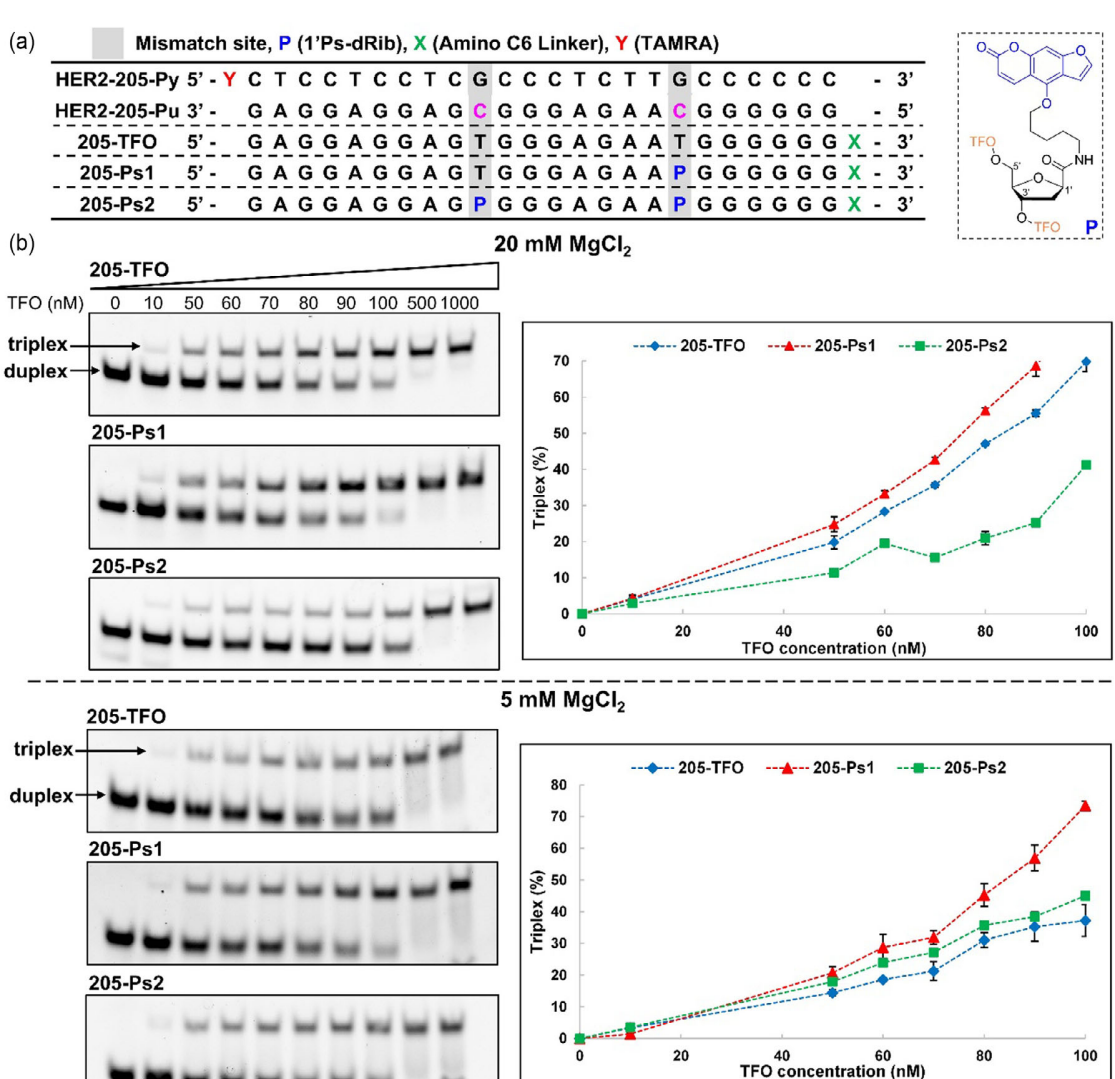
Analysis of the triplex formation with HER2‐205 sequence using Native PAGE. a) Sequences of target DNA duplexes and TFOs. b) Representative gel images of each TFOs with target duplex and quantification results of Native PAGE, which are displayed as the mean ± standard deviation (s.d.) for *n* = 3 replicates.

To increase the binding affinity of the TFO for the target duplex, we need to address the intrinsic problems of the TFO technology, which is the requirement of a consecutive purine base sequence (polypurine sequence) in one strand of the target duplex DNA, as the Hoogsteen hydrogen bonding is formed only between the TFO and the purine bases of the duplex DNA. When we try to find the potential triplex formation site (polypurine sequence) in the target gene, the candidate sequences often contain one or two pyrimidine bases that are often mentioned as a mismatch site, which causes a reduction in the binding affinity of TFO for the target sequence. To solve this problem, several artificial nucleobase analogs have been developed that can form hydrogen bonds with pyrimidine bases within the polypurine sequence. In addition, the introduction of DNA intercalators into TFOs has been reported to effectively improve the binding affinity of TFO for target duplex.^[^ ^6^ ^]^ Recently, our group developed the novel nucleoside analog **P** (**Figure** **2**) that possesses a psoralen, a DNA intercalator, at its 1'‐position. We successfully demonstrated that the replacement of the mismatch bases of the parallel TFO with **P**, named OPTO after “1´(One)‐psoralen‐conjugated TFO,” improves the binding affinity of the parallel TFO for the target duplex.^[^ ^7^ ^]^

In this study, we applied our novel nucleoside analog **P** to antiparallel TFO to increase the binding affinity of the TFO for amplified HER2 gene loci and evaluated the apoptosis induction of HER2‐amplified breast cancer cells (Figure 1). Our study shed light on the relationships between the binding affinity of the TFO for amplified gene loci under physiological conditions and the apoptosis induction levels, indicating the efficacy of DNA intercalator‐conjugated TFO for anticancer therapy.

## 2 Results and Discussion

### 2.1 Evaluation of the Thermodynamic Stability of Triplexes with HER2 Gene Sequences

The phosphoramidite for the nucleotide analog **P** was synthesized according to our recent report.^[^ ^7^ ^]^ OPTOs were synthesized at Ajinomoto Genedesign (Osaka, Japan). Briefly, the phosphoramidite for **P** was introduced into oligonucleotides on the CPG support using the phosphoramidite chemistry (Figure S1–4, Supporting Information). According to the Rogers’ report,^[^ ^5^ ^]^ a sequence within the HER2 gene (HER2‐205) was chosen as a target for OPTO (Figure 2a). As this target sequence contains two mismatch sites, the replacement of these two mismatch bases with **P** would be expected to increase the binding affinity for the target duplex. Therefore, we prepared two types of OPTOs with one or two **P** (205‐Ps1 and 205‐Ps2, respectively). We evaluated the binding affinity of the OPTOs for the HER2‐205 sequence using nondenaturing polyacrylamide gel electrophoresis (Native PAGE). In this experiment, the 5´ end of the pyrimidine base‐rich sequence (HER2‐205Py) was labeled with tetramethylrhodamine. Further, to improve the anticancer activity, an amino linker was introduced at the 3´ end of TFOs, which has been reported to improve the cellular uptake and nuclear translocation of TFOs.^[^ ^8^ ^]^ Sample solutions containing different concentrations of TFOs were prepared and analyzed by Native PAGE, and the fluorescence intensity of the DNA bands was quantified (Figure 2b). It is well known that the magnesium ion (Mg^2+^) is important in the formation of a stable triplex by mitigating the electrostatic repulsion of the phosphate backbone of oligonucleotides. For the TFO binding assay using Native PAGE, the concentration of Mg^2+^ in the sample solution generally varies (2.5–20 mM).^[^ ^9^ ^]^ However, the conformational difference of the triplex depending on the Mg^2+^ concentration might cause either the promotion or interference of the intercalation of the psoralen moiety in the TFO, leading either to the stabilization or destabilization of the corresponding triplex.Therefore, we conducted this experiment at two different Mg^2+^ concentrations (5 and 20 mM). The obtained representative gel images for each TFO (205‐TFO, 205‐Ps1, and 205‐Ps2) at both 5 and 20 mM Mg^2+^ concentrations are shown in Figure 2b (Figure S5 and Table S1, Supporting Information). In each gel, the band in the first lane (TFO concentration is 0 nM) stands for duplex. The band transition from duplex to triplex was observed depending on the TFO concentration. The graphical charts in Figure 2b show the quantification results of the DNA bands in the obtained gels. At 20 mM Mg^2+^ concentration, the unmodified 205‐TFO showed a higher binding affinity for the target duplex than that observed at 5 mM Mg^2+^. The 205‐Ps1 showed a higher binding affinity for the target duplex than the unmodified 205‐TFO and 205‐Ps2 at both Mg^2+^ concentrations, suggesting the triplex stabilization effect of **P**. In contrast, the additional introduction of **P** in the 205‐Ps2 decreased the binding affinity of 205‐Ps2 for the target duplex compared than the binding affinity of 205‐Ps1 for the same target sequence, although it was still higher than that of the unmodified 205‐TFO at 5 mM Mg^2+^.

**Figure 3:**
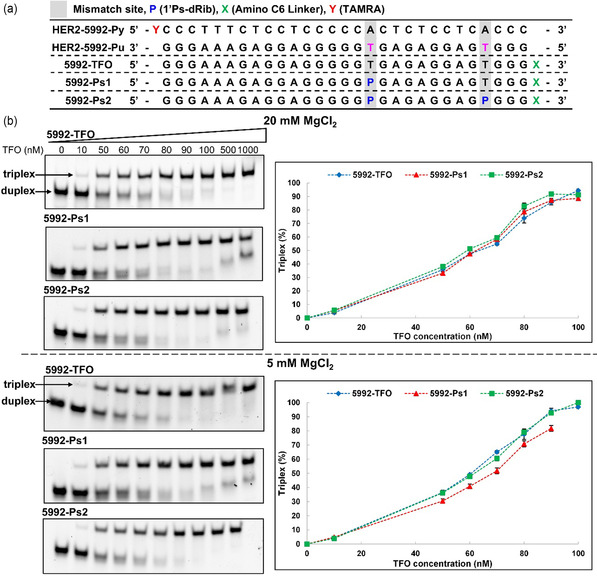
Analysis of the triplex formation with HER2‐5992 sequence using Native PAGE. a) Sequences of target DNA duplexes and TFOs. b) Representative gel images of each TFOs with target duplex and quantification results of Native PAGE, which are displayed as the mean ± standard deviation (s.d.) for *n* = 3 replicates.

We also conducted the same experiment using another sequence (HER2‐5992) (**Figure** **3**a, S5, and Table S1, Supporting Information) that was also the target site of TFO in the Rogers’ report.^[^ ^5^ ^]^ In this case, 5992‐Ps2 showed a higher binding affinity for the target duplex than did 5992‐TFO and 5992‐Ps1 at 20 mM Mg^2+^ concentration, whereas 5992‐TFO showed a higher binding affinity for the target duplex than 5992‐Ps1 at 5 mM Mg^2+^ concentration. This result also suggests that the replacement of mismatch bases in TFO with **P** does not always increase the binding affinity for the target duplex, and it depends on both the Mg^2+^ concentration and the target sequences.

The binding association constants of the TFOs (*K*_a_) were calculated according to the literature (**Table** **1** and Figure S6 and 7, Supporting Information).^[9d,e]^ These *K*_a_ values also suggest that the DNA intercalator causes either stabilization or destabilization of the corresponding triplex depending on the Mg^2+^ concentration and target DNA sequence.

**Table 1:** *K*_a_ values of TFOs.

	*K*_a_ value (10^6^ M^−1^) at 20 mM Mg^2+^	*K*_a_ value (10^6^ M^−1^) at 5 mM Mg^2+^
205‐TFO	30	14
205‐Ps1	40	30
205‐Ps2	8	18
5992‐TFO	77	107
5992‐Ps1	87	58
5992‐Ps2	121	93

### 2.2 Evaluation of the Anticancer Activities of TFOs Against HER2‐Positive Breast Cancer Cells

**Figure 4:**
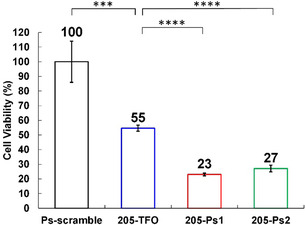
The anticancer activities of TFOs against HER2‐positive breast cancer cells (BT474) are displayed as the mean ± standard deviation (s.d.) for *n* = 4 replicates. The final concentration of TFOs was 25 nM. Statistical significance determined by two‐tailed *t*‐test. ***P < 0.001, ****P < 0.0001.

As the 205‐Ps1 showed higher binding affinity for the HER2‐205 sequence in a wide range of Mg^2+^ concentration conditions, we hypothesized that this higher binding affinity of 205‐Ps1 for the target sequence would lead to stronger cell death of HER2‐positive breast cancer cells (BT474). Thus, we evaluated the anticancer activities of these TFOs (205‐TFO, 205‐Ps1, and 205‐Ps2) against BT474 cells (**Figure** **4** and Table S2, Supporting Information). We also prepared a scramble sequence (Ps‐scramble, Figure S8, Supporting Information) as a negative control. The TFOs were introduced in the cancer cells by lipofection, and cell viability was analyzed using the WST‐8 assay. The unmodified 205‐TFO induced significant cell death of HER2‐positive breast cancer cells, as demonstrated in the Rogers’ report.^[^ ^5^ ^]^ As expected, the cell death response induced by 205‐Ps1 was the strongest among the TFOs, suggesting that the binding affinity of the TFO for the target HER2 gene was related to anticancer activities. These results are in good accordance with the *K*_a_ values obtained at 5 mM Mg^2+^ concentration. When we consider the endogenous Mg^2+^ concentration range of a mammalian cell (0.5–10 mM),^[^ ^10^ ^]^ it is reasonable to think that the anticancer activities of the TFOs were related to the *K*_a_ values obtained at 5 mM Mg^2+^ concentration. This suggests the importance of evaluating the binding affinity of the TFO for the target duplex within the Mg^2+^ concentration range of a mammalian cell (0.5–10 mM), especially for the DNA intercalator‐conjugated TFO, as the intercalation of the DNA intercalator was affected by the conformational changes in the triplex caused by Mg^2+^ concentration and target DNA sequence, resulting in either the stabilization or destabilization of the corresponding triplex.

## 3 Conclusions

The binding affinity of our DNA intercalator‐conjugated TFOs (OPTO) for the target duplex was evaluated using two target sequences in the HER2 gene (HER2‐205 and HER2‐5992). In the case of TFO 205‐Ps1, in which a psoralen was replaced with one mismatched base of 205‐TFO, the binding affinity for the target duplex was improved. However, we found that this stabilization effect of OPTO is completely dependent on the sequence and Mg^2+^ concentration, possibly causing the conformational differences of the triplex. As we expected, 205‐Ps1, which has a higher affinity with the Her2‐205 target sequence, had a stronger anticancer activity against BT‐474 (one of the HER2‐positive breast cancer cell) than unmodified 205‐TFO. These results suggest that the enhanced binding affinity of the TFO for the target duplex structure leads to a stronger cell death and that OPTO may have potent anticancer activity against various HER2 copy number‐amplified breast cancer cells other than BT‐474.

## 4 Experimental Section

4.1

4.1.1

##### Solid‐Phase Synthesis of Modified TFOs with P (205‐Ps1 and 205‐Ps2, 5992‐Ps1, and 5992‐Ps2)

The synthesis of 205‐Ps1 and 205‐Ps2, 5992‐Ps1, and 5992‐Ps2 were performed at Ajinomoto Genedesign. Briefly, the phosphoramidites were introduced to oligonucleotides on the CPG support using phosphoramidite chemistry, and the purification of the oligos was performed by reverse‐phased HPLC (RP‐HPLC) on an X‐Bridge C18 column (2.5 μm, 4.6 * 75 mm, Waters, Co. Ltd., MA, USA) with a linear gradient of methanol in 100 mM 1,1,1,3,3,3‐hexafluoro‐2‐propanol containing 8 mM triethylamine at a flow rate of 1.0 mL min^−1^ at 60 °C (methanol gradient: 5–30%, 20 min). The mass spectra were obtained using MALDI‐TOF‐MS (Bruker DALTONICS autoflex speed).

##### Triplex Formation Analysis using Nondenaturing Polyacrylamide Gel Electrophoresis (Native PAGE)

Sample solutions containing the ds‐DNAs (100 nM) and different concentrations of TFOs (0, 10, 50, 60, 70, 80, 90, 100, 500, and 1000 nM each), 20 mM Tris‐HCl (pH 7.0), and 5 mM MgCl_2_ were denatured by heating to 95 °C and subsequently cooling to 4 °C at 0.5 °C min^−1^ and were then incubated at 37 °C for 12 h. Then, the samples were diluted with 40 wt% sucrose aq (sample: 40 wt% sucrose aq = 1:4 v v^−1^) and analyzed with 20% native polyacrylamide gel (PAGE) containing 5 mM Mg^2+^ in TBM (37 °C, 120 V, 90 min). The DNA bands were stained by SYBR Gold, and then the gels were transferred to imaging plates. The gel images were analyzed and quantitated using ChemDoc Touch MP (BioRad, CA, USA).

##### Evaluation of Anticancer Activities of TFO using the WST‐8 Assay

The BT‐474 cells were planted in a 96‐well plate (1 × 10^4^ cells/well) and incubated for 24 h (5% CO_2_, 37 ºC). Then, the cells were transfected with each TFO (25 nM) using lipofectamine 3 000 (Thermo Scientific, MA, USA). After 24 h incubation (5% CO_2_, 37 ºC), the cellular viability was quantified by the WST‐8 assay using the Cell Counting Kit‐8 (DOJINDO, Kumamoto, Japan), and the absorbance at 450 nm was measured using a multimode plate reader Cytation3 (BioTek, CA, USA).

## Conflict of Interest

The authors declare no conflict of interest.

## Supporting information

Supplementary Material

## Data Availability

The data that support the findings of this study are available in the supplementary material of this article.
