# Supplementary material for: Efficacy of DNA Intercalator‐Conjugated Triplex‐Forming Oligonucleotide as Anticancer Agent
Source: ChemMedChem. 2025 Sep 14;20(20):e202500325. doi: 10.1002/cmdc.202500325 (PMC12530838; doi:10.1002/cmdc.202500325)
Supplement: Supplementary file 1 — Supplementary Material [file CMDC-20-e202500325-s001.pdf]

Contents

General methods 2

Figure S1. Analytical data of 205-Ps1. 3

Figure S2. Analytical data of 205-Ps2. 4

Figure S3. Analytical data of 5992-Ps1 5

Figure S4. Analytical data of 5992-Ps2 6

Figure S5. The whole gel images. 8

Figure S6. The details of K_a value calculation using datas collected for 205-Ps1 11

Figure S7. The calibration curves for the K_a value calculation were shown. 12

Table S1. Quantitative analysis of the obtained gels. 14

Table S2. The row datas of WST-8 assay (absorbance at 450 nm). 20

Figure S8. The sequence of Ps-scramble. 21

General methods

The phosphoramidite of P was synthesized according to our previous report [ref. 1]. The target duplexes (HER2-205-Py and HER2-205-Pu, HER2-5992-Py, and HER2-5992-Pu) and unmodified TFOs (205-TFO and 5922-TFO) were purchased from FASMAC Co., Ltd. (Kanagawa, Japan). The solid-phase syntheses of the modified TFOs with P (205-Ps1 and 205-Ps2, 5992-Ps1, and 5992-Ps2) were performed at Ajinomoto Genedesign (Osaka, Japan). The fluorescence intensity of the DNA bands in the gel was quantified using ImageJ.

Ref. 1

Mikame, Y.; Toyama, H.; Dohno, C.; Wada, T.; Yamayoshi, A. Development and functional evaluation of a psoralen-conjugated nucleoside mimic for triplex-forming oligonucleotides. *Commun. Chem*. **2025**, *8*, 18.

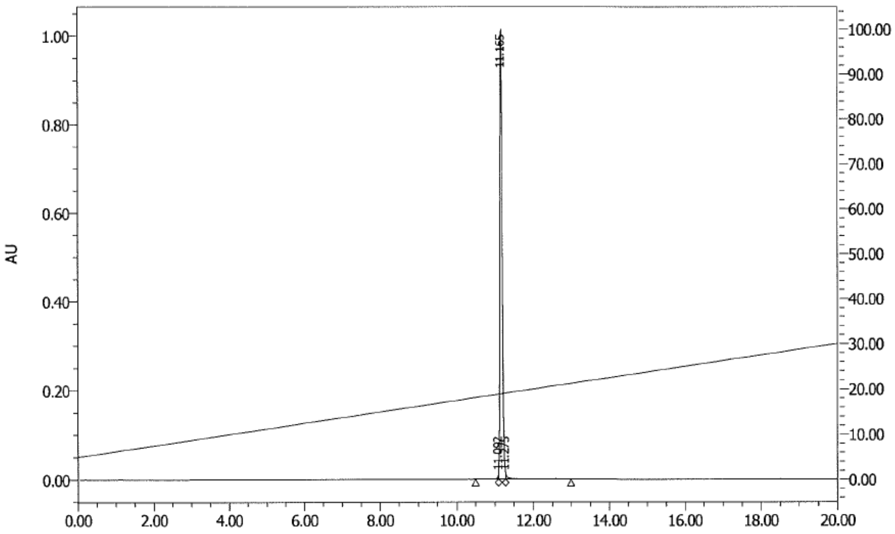

Methanol gradient: 5-30%, retention time: 11.2 min.

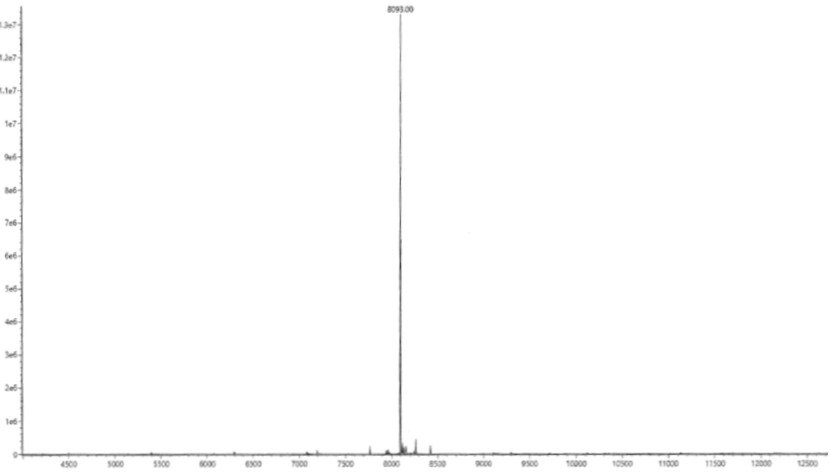

Calcd for $[C_{258}H_{316}N_{114}O_{146}P_{24}]^{-}$: 8093.4 found 8093.0.

Figure S1. Analytical data of 205-Ps1. HPLC analysis and MALDI-MS spectra.

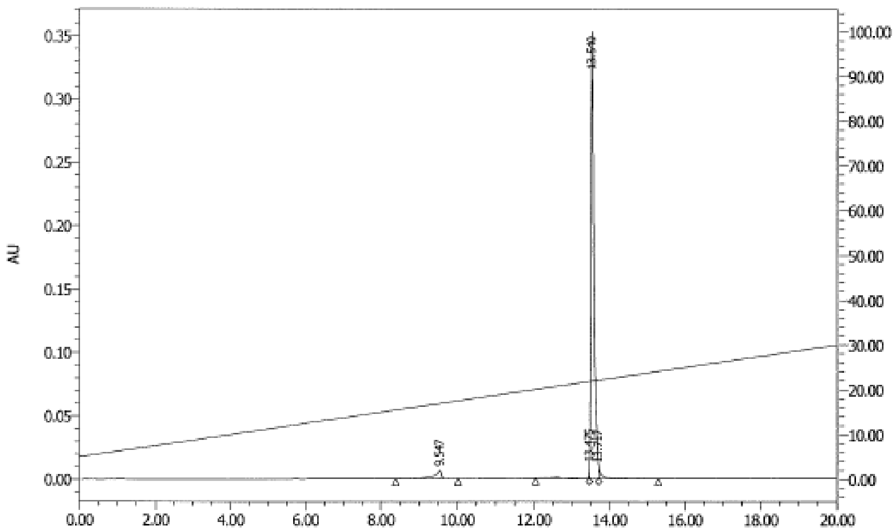

Methanol gradient: 5-30%, retention time: 13.5 min.

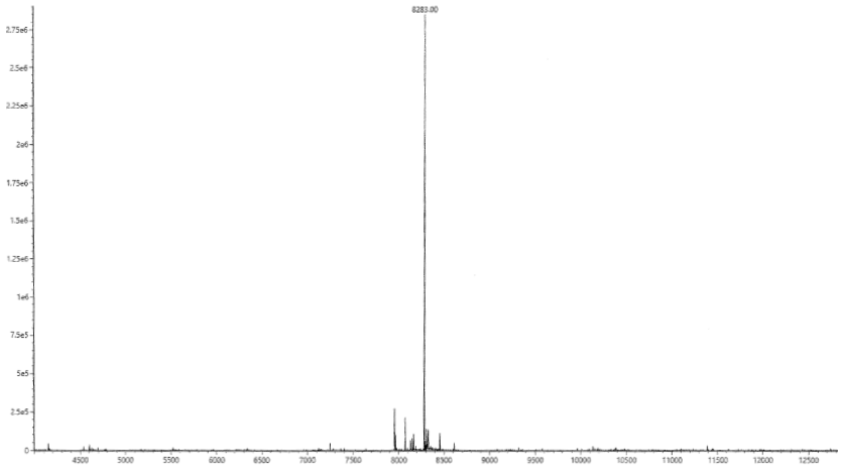

Calcd for $[C_{270}H_{327}N_{113}O_{149}P_{24}]^-$: 8282.6 found 8283.0.

Figure S2. Analytical data of 205-Ps2. HPLC analysis and MALDI-MS spectra.

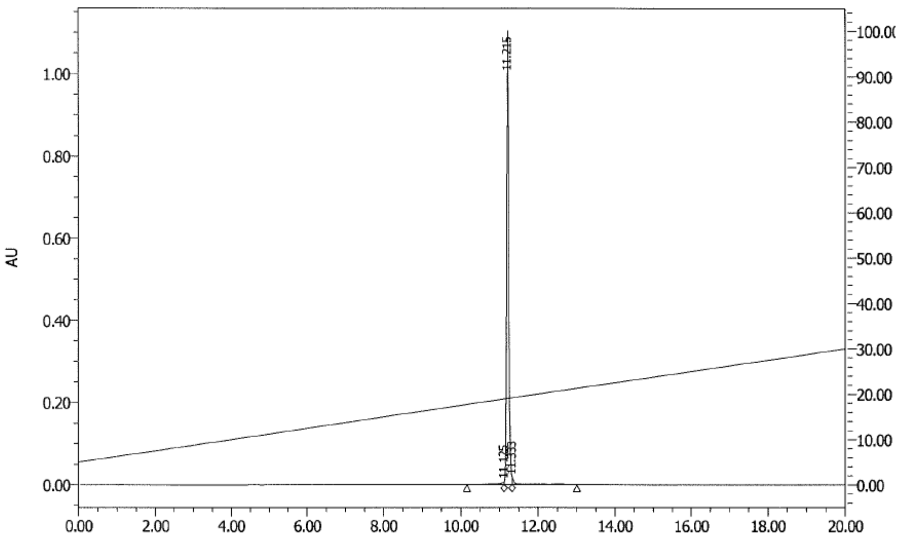

Methanol gradient: 5-30%, retention time: 11.2 min.

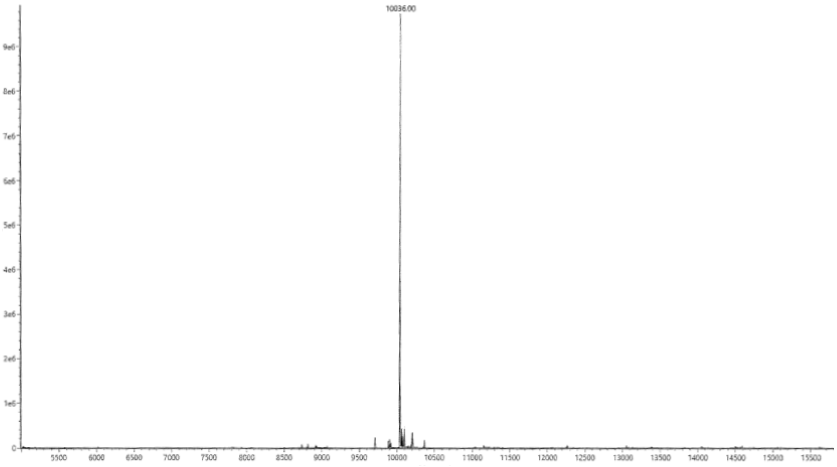

Calcd for $[C_{318}H_{388}N_{144}O_{180}P_{30}]^{-}$: 10036.7 found 10036.0.

Figure S3. Analytical data of 5992-Ps1. HPLC analysis and MALDI-MS spectra.

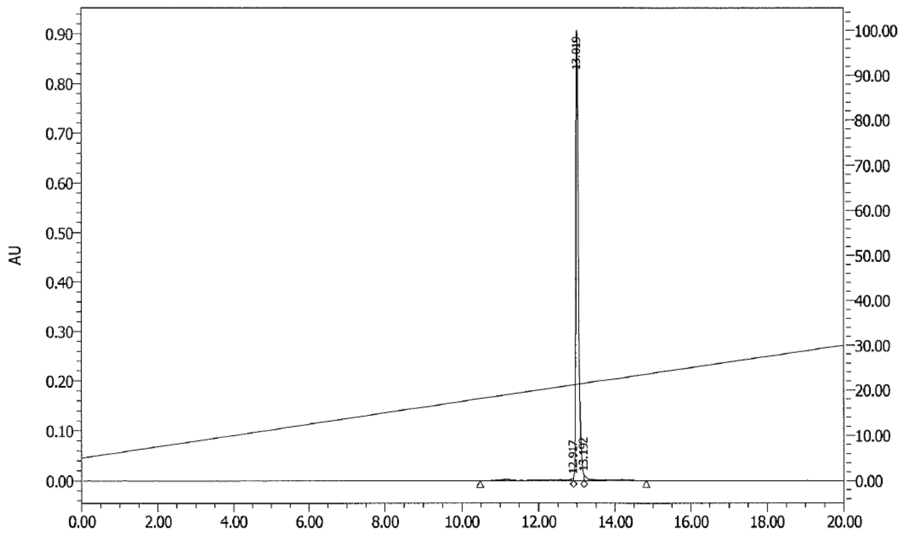

Methanol gradient: 5-30%, retention time: 13.0 min.

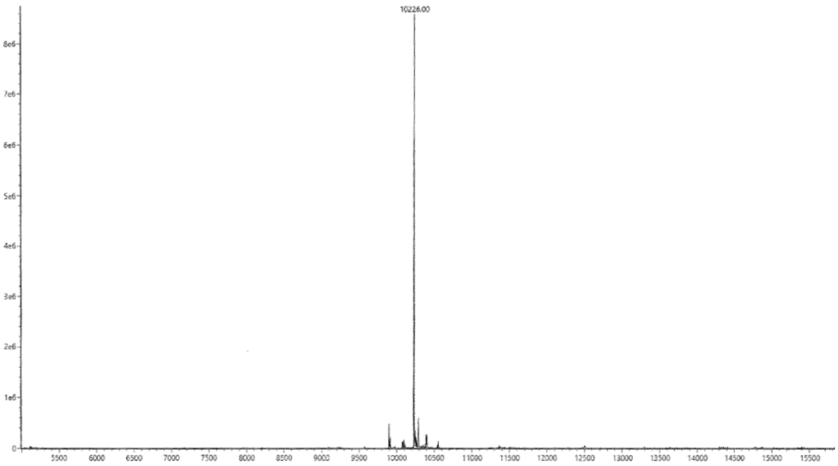

Calcd for $[C_{330}H_{399}N_{143}O_{183}P_{30}]^{-}$: 10225.9 found 10226.0.

Figure S4. Analytical data of 5992-Ps2. HPLC analysis and MALDI-MS spectra.

The gel image of 205-TFO

20 mM $MgCl_2$

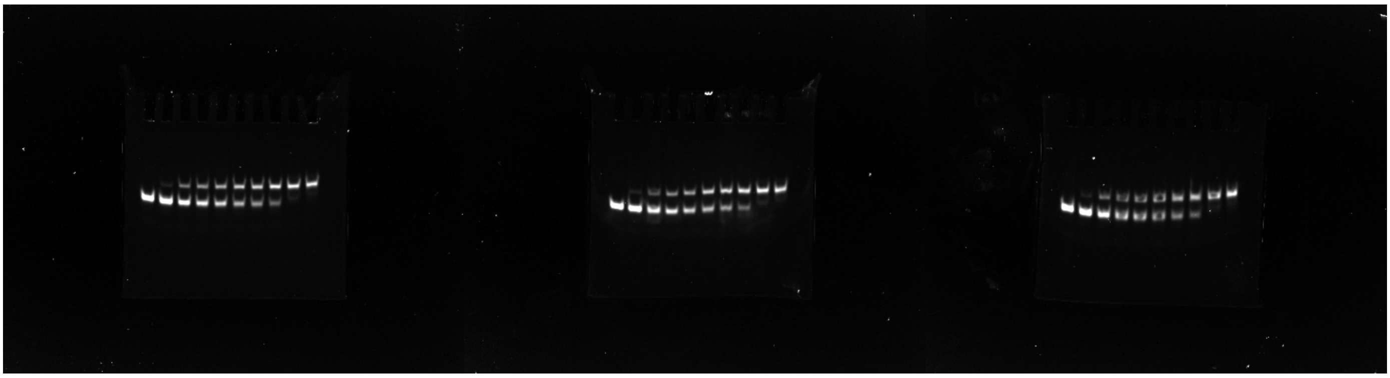

5 mM $MgCl_2$

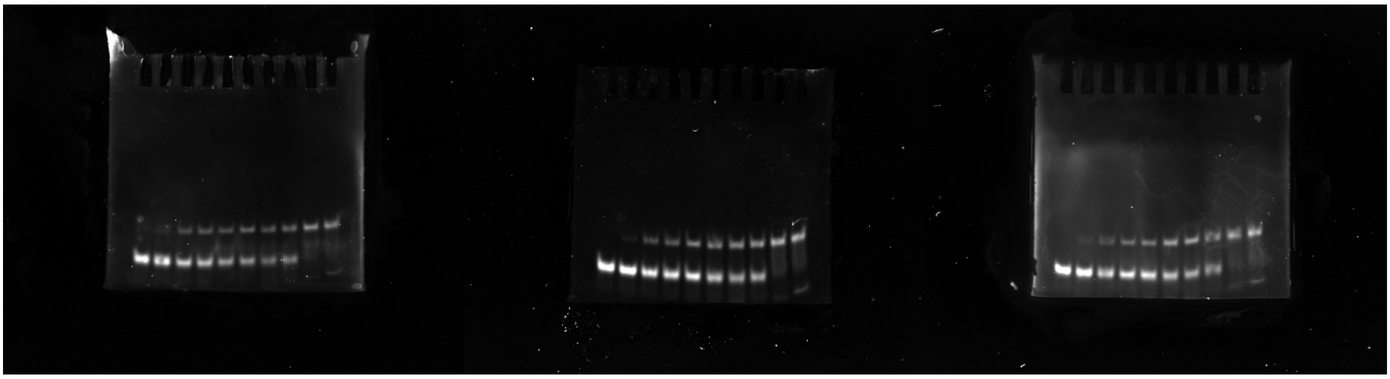

The gel image of 205-Ps1

20 mM $MgCl_2$

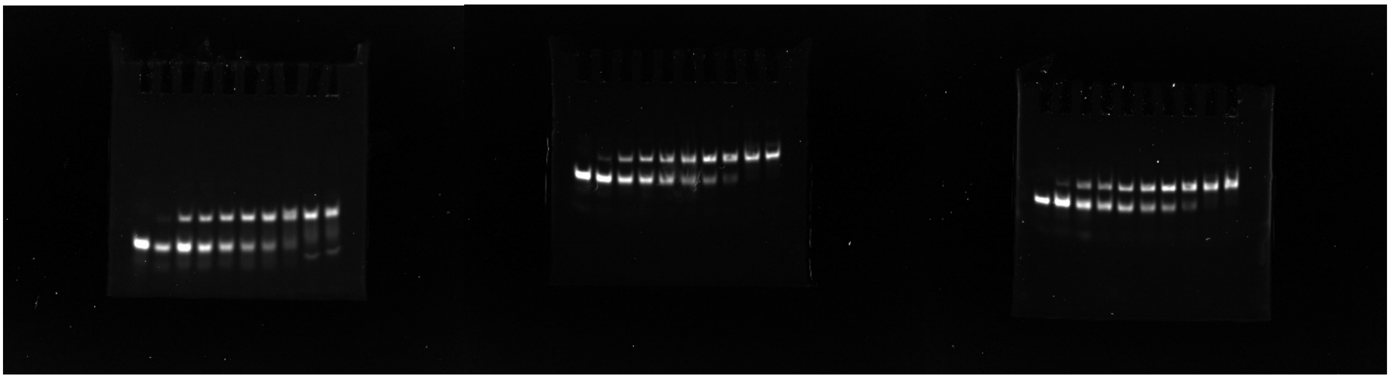

5 mM $MgCl_2$

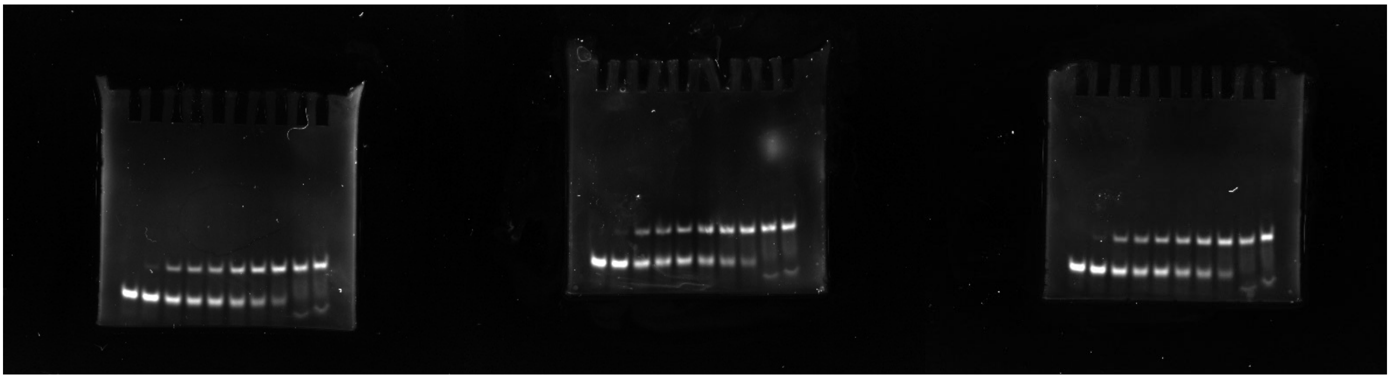

The gel image of 205-Ps2

20 mM $MgCl_2$

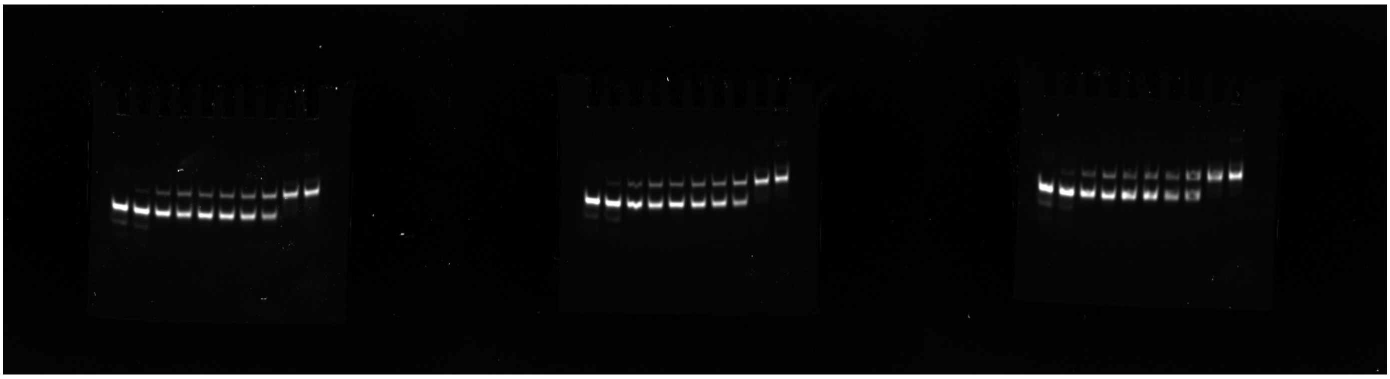

5 mM $MgCl_2$

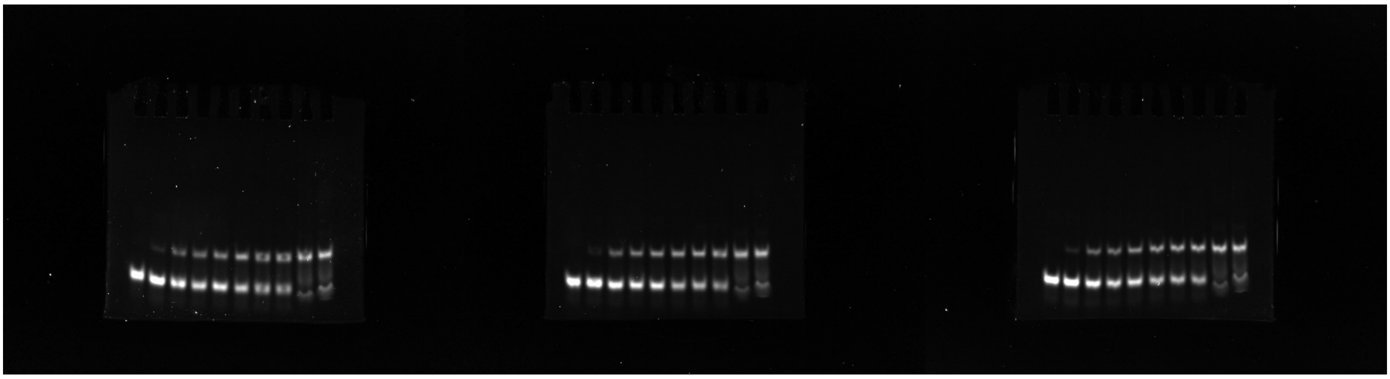

The gel image of 5992-TFO

20 mM $MgCl_2$

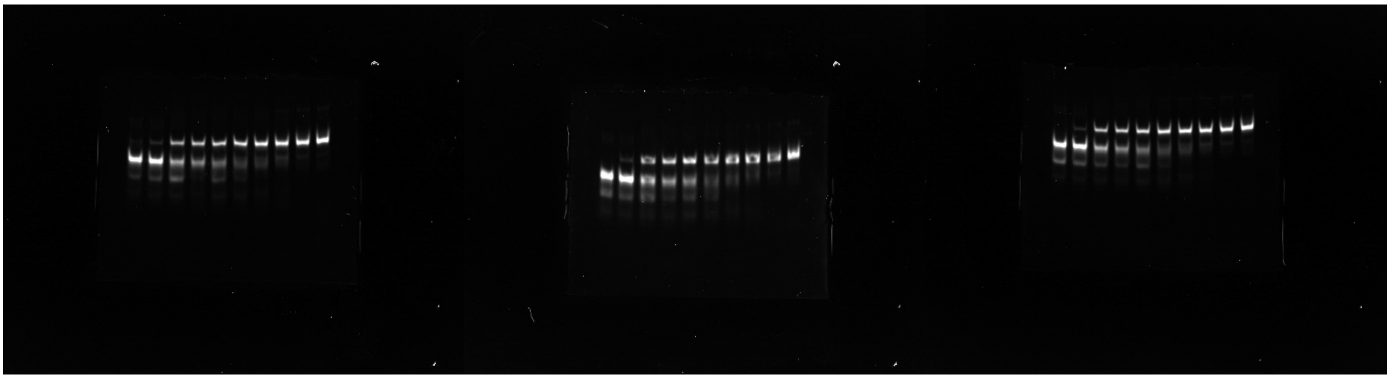

5 mM $MgCl_2$

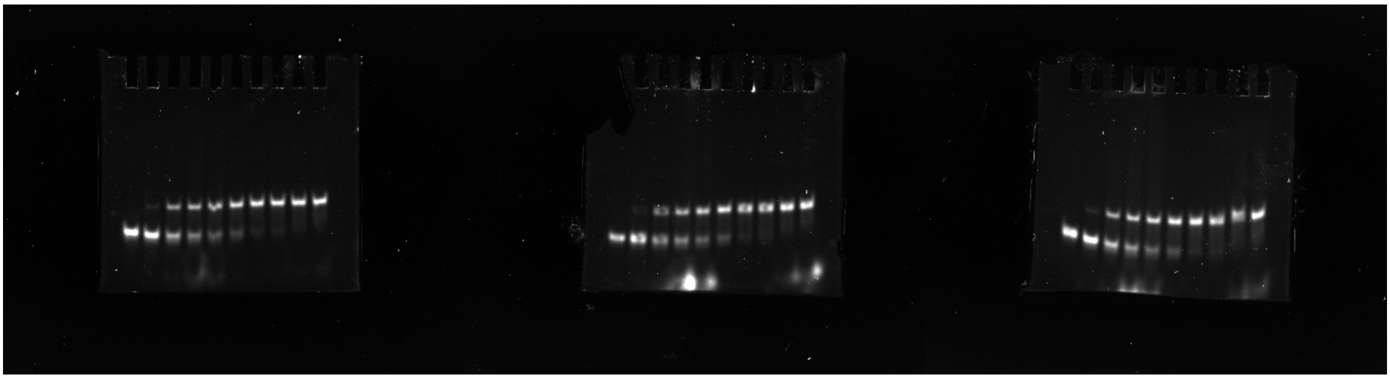

The gel image of 5992-Ps1

20 mM $MgCl_2$

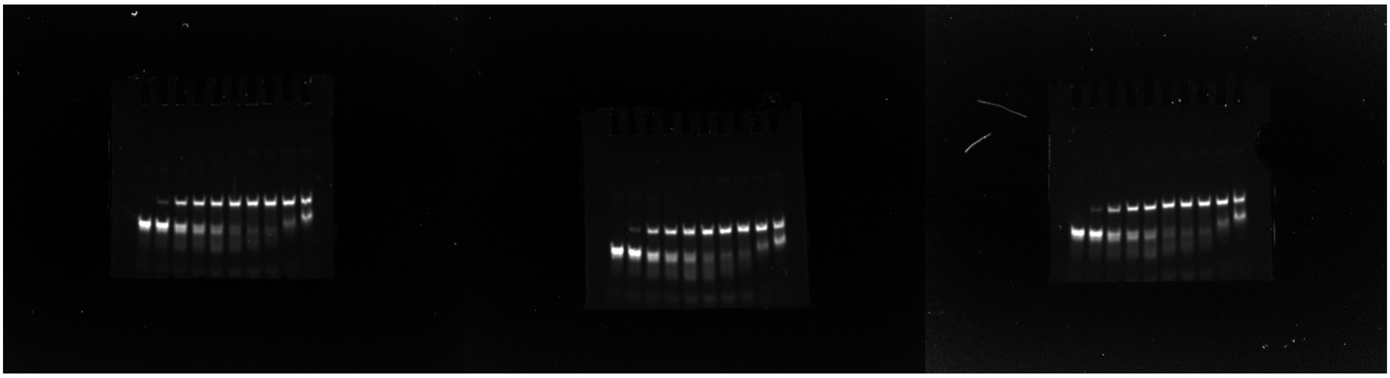

5 mM $MgCl_2$

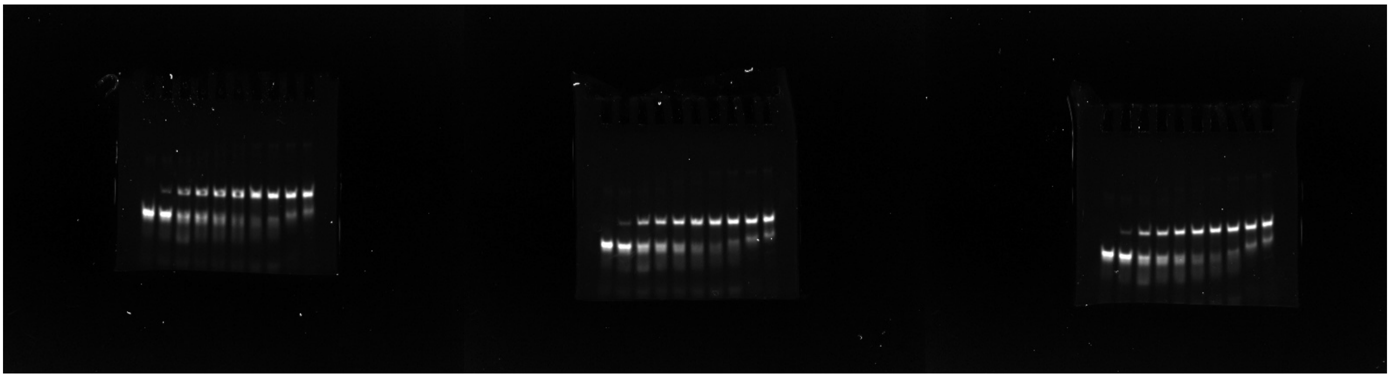

The gel image of 5992-Ps2

20 mM $MgCl_2$

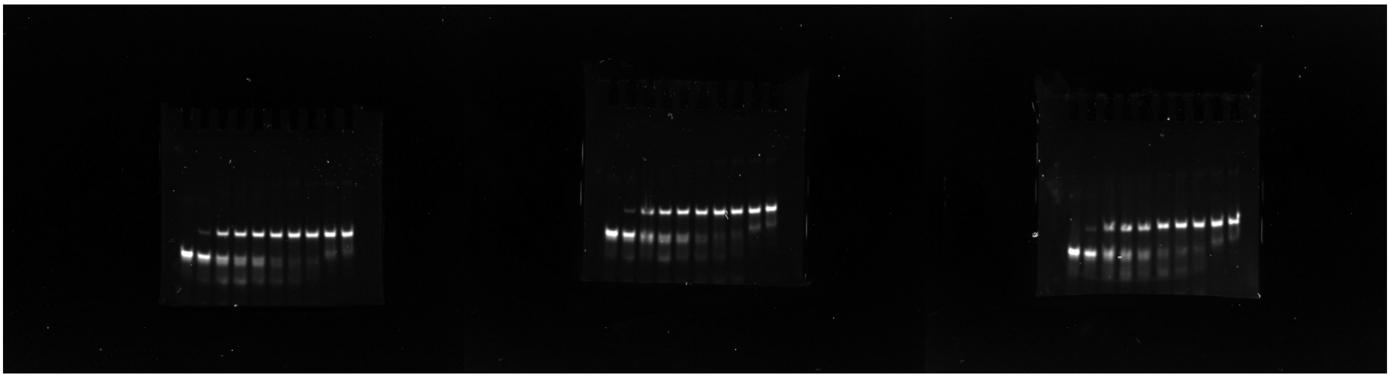

5 mM $MgCl_2$

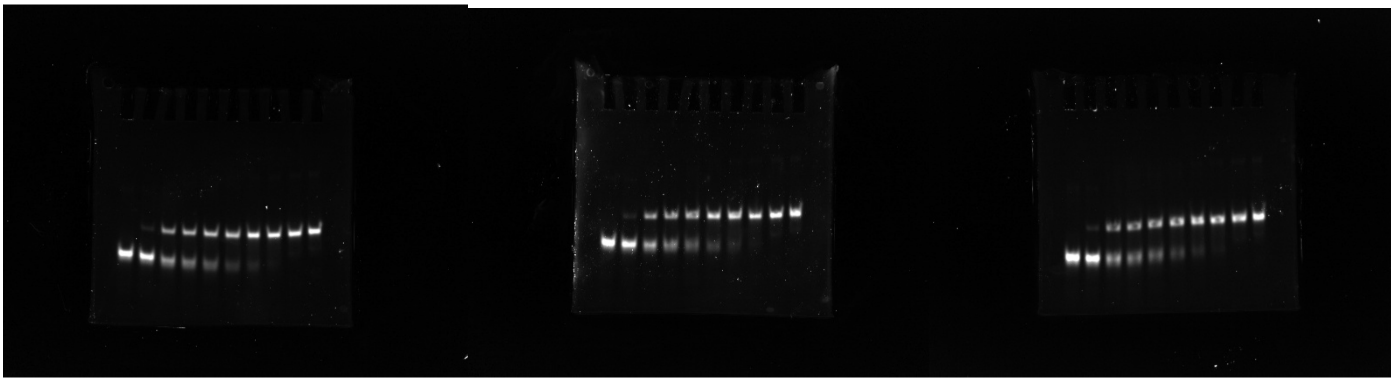

Figure S5. The whole gel images.

1) $K_a\ (10^6\ M^{-1}) = [Triplex]/([free\ TFO][free\ Duplex])$
2) $[Triplex] = [free\ Duplex]$
3) $[free\ TFO] = [initial\ TFO] - [Triplex]$
4) $K_a = 1/[free\ TFO]$

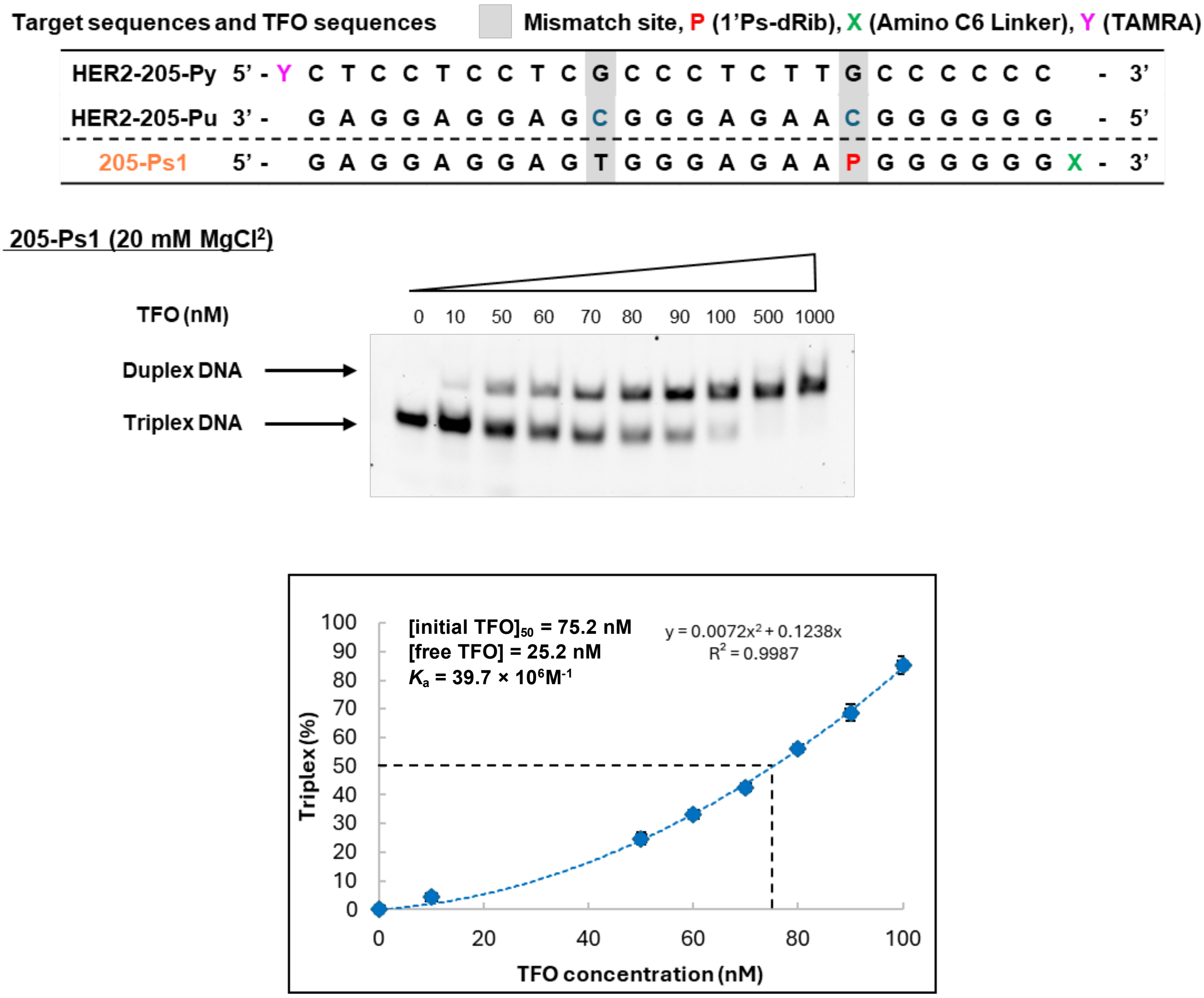

Figure S6. The details of K_a value calculation using datas collected for 205-Ps1. The relationships between the yields of formed triplex bands and the concentration of TFO were shown in the graph. From this graph, the initial TFO concentration ($[initial\ TFO]_{50}$) to form 50 % triplex was calculated from the calibration curve, then K_a values were determined sccording to equation (1)-(4) (ref. 2, 3).

Ref. 2

H. Okamura, Y. Taniguchi, S. Sasaki, *ChemBioChem*. **2014**, *15*, 2374-2378.

Ref. 3

H. Okamura, Y. Taniguchi, S. Sasaki, *Angew. Chem. Int. Ed*. **2016**, *55*, 12445-12449.

HER2-205-sequence at 20 mM $MgCl_2$

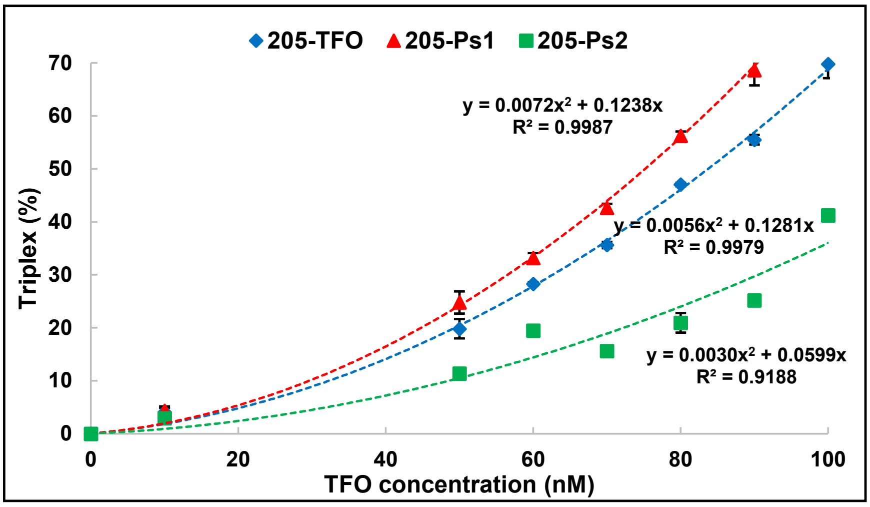

HER2-205-sequence at 5 mM $MgCl_2$

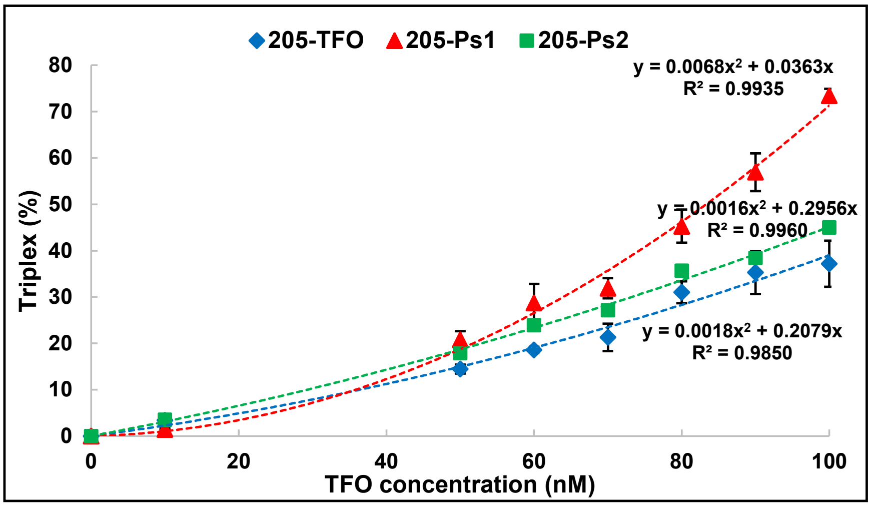

HER2-5992-sequence at 20 mM $MgCl_2$

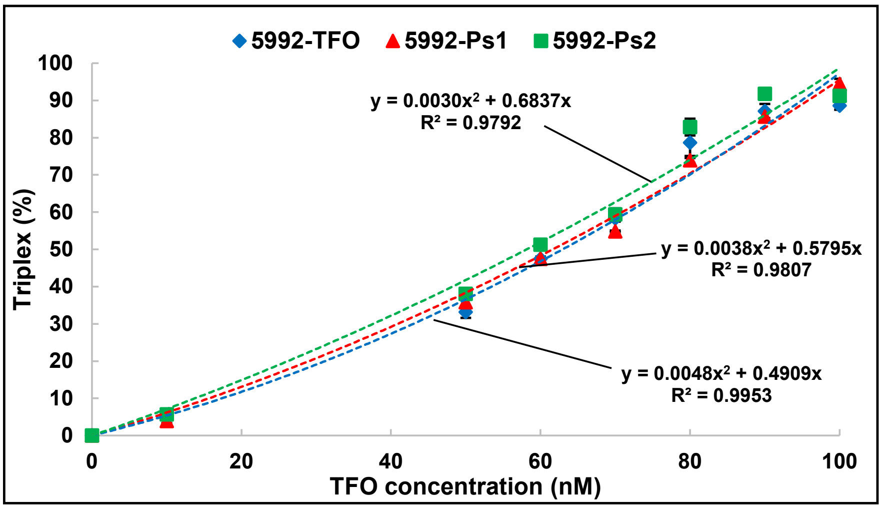

HER2-5992-sequence at 5 mM $MgCl_2$

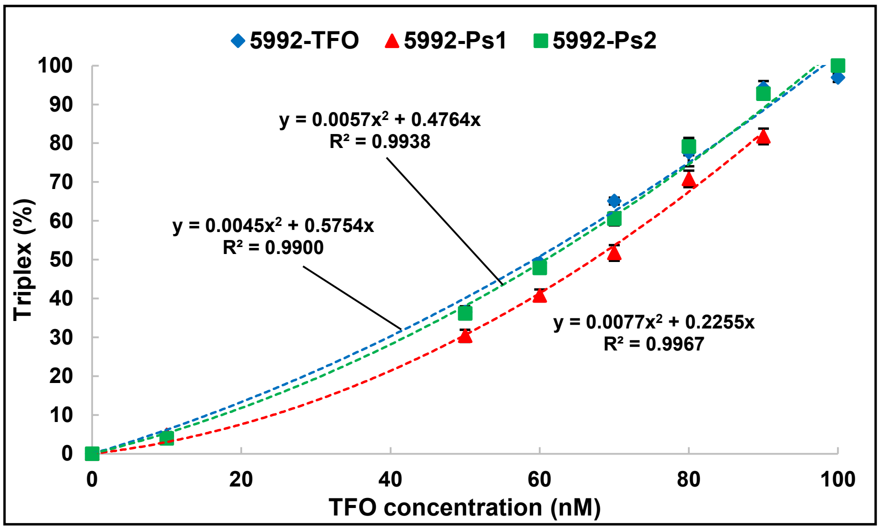

Figure S7. The calibration curves used for the K_a value calculation were shown. The initial TFO concentration ([initial TFO]$_{50}$) to form 50 % triplex was calculated from each calibration curve.

Table S1. Quantitative analysis of the obtained gels.

Quantitative analysis of 205-TFO

20 mM $MgCl_2$

TFO concentration (nM)	0	10	50	60	70	80	90	100
Triplex (%)	**0**	**3**	**21**	**28**	**36**	**47**	**55**	**69**
Duplex (Area)	221118	38438	28008	24043	21322	15560	12697	8674
Triplex (Area)	0	1346	7419	9561	12028	13798	15518	19357

TFO concentration (nM)	0	10	50	60	70	80	90	100
Triplex (%)	**0**	**4**	**21**	**28**	**36**	**47**	**57**	**73**
Duplex (Area)	221414	36811	30375	23878	19131	15369	11762	5742
Triplex (Area)	0	1491	7966	9510	10691	13464	15316	15326

TFO concentration (nM)	0	10	50	60	70	80	90	100
Triplex (%)	**0**	**5**	**18**	**28**	**35**	**47**	**55**	**68**
Duplex (Area)	221291	34840	29124	22230	17862	15939	13520	9040
Triplex (Area)	0	1807	6261	8624	9594	14374	16507	18840

5 mM $MgCl_2$

TFO concentration (nM)	0	10	50	60	70	80	90	100
Triplex (%)	**0**	**4**	**13**	**18**	**19**	**33**	**38**	**42**
Duplex (Area)	221414	29746	22487	24508	24690	17135	15049	12653
Triplex (Area)	0	1386	3479	5429	5745	8287	9321	8999

TFO concentration (nM)	0	10	50	60	70	80	90	100
Triplex (%)	**0**	**2**	**15**	**19**	**25**	**32**	**38**	**38**
Duplex (Area)	33606	28723	23195	23195	21669	19109	15212	15758
Triplex (Area)	0	545	3978	5581	7051	9052	9192	9732

TFO concentration (nM)	0	10	50	60	70	80	90	100
Triplex (%)	**0**	**4**	**15**	**18**	**20**	**28**	**30**	**32**
Duplex (Area)	221426	28984	19395	20410	15348	13674	11779	13132
Triplex (Area)	0	1150	3502	4554	3937	5398	5032	6122

Quantitative analysis of 205-Ps1

20 mM $MgCl_2$

TFO concentration (nM)	0	10	50	60	70	80	90	100
Triplex (%)	**0**	**5**	**26**	**33**	**42**	**56**	**72**	**87**
Duplex (Area)	221406	35258	26252	22672	20053	14329	8050	2941
Triplex (Area)	0	1910	9205	11241	14728	18051	20384	19842

TFO concentration (nM)	0	10	50	60	70	80	90	100
Triplex (%)	**0**	**4**	**26**	**34**	**43**	**57**	**68**	**87**
Duplex (Area)	220908	44714	31230	24353	22434	16845	11587	3757
Triplex (Area)	0	2002	10981	12611	17257	22518	25169	26006

TFO concentration (nM)	0	10	50	60	70	80	90	100
Triplex (%)	**0**	**3**	**22**	**32**	**42**	**56**	**66**	**82**
Duplex (Area)	220651	21907	32337	24643	19289	12605	9045	4032
Triplex (Area)	0	789	9296	11722	13983	15954	17452	18121

---

5 mM $MgCl_2$

TFO concentration (nM)	0	10	50	60	70	80	90	100
Triplex (%)	**0**	**1**	**21**	**31**	**31**	**42**	**52**	**72**
Duplex (Area)	221416	27812	21072	19026	19682	15294	12691	6466
Triplex (Area)	0	373	5496	8368	8997	10888	13949	16519

TFO concentration (nM)	0	10	50	60	70	80	90	100
Triplex (%)	**0**	**2**	**19**	**24**	**30**	**45**	**60**	**74**
Duplex (Area)	221411	29638	21730	19005	17926	15331	9926	6338
Triplex (Area)	0	474	5051	6022	7693	12789	14984	17640

TFO concentration (nM)	0	10	50	60	70	80	90	100
Triplex (%)	**0**	**1**	**23**	**32**	**34**	**49**	**58**	**75**
Duplex (Area)	221408	31096	22352	19068	18504	15808	10904	6270
Triplex (Area)	0	426	6533	8816	9640	15045	15224	18682

<u>Quantitative analysis of 205-Ps2</u>

20 mM $MgCl_2$

TFO concentration (nM)	0	10	50	60	70	80	90	100
Triplex (%)	**0**	**3**	**12**	**19**	**16**	**20**	**26**	**42**

Duplex (Area)	221420	31489	27090	24398	26822	23964	15320	14938
Triplex (Area)	0	1013	3756	5894	5190	5973	5356	10923

TFO concentration (nM)	0	10	50	60	70	80	90	100
Triplex (%)	**0**	**3**	**11**	**20**	**15**	**23**	**25**	**41**
Duplex (Area)	32823	29759	26224	22538	24204	21208	19298	12844
Triplex (Area)	0	909	3282	5602	4439	6351	6584	8920

TFO concentration (nM)	0	10	50	60	70	80	90	100
Triplex (%)	**0**	**3**	**11**	**19**	**15**	**20**	**24**	**41**
Duplex (Area)	221212	28323	23882	20947	23990	22028	19288	13545
Triplex (Area)	0	751	2877	4905	4233	5423	6127	9231

---

5 mM $MgCl_2$

TFO concentration (nM)	0	10	50	60	70	80	90	100
Triplex (%)	**0**	**4**	**19**	**24**	**27**	**35**	**39**	**44**
Duplex (Area)	221051	29010	24194	19790	20029	16721	16215	14245
Triplex (Area)	0	1221	5500	6235	7444	8983	10325	11392

TFO concentration (nM)	0	10	50	60	70	80	90	100
Triplex (%)	**0**	**3**	**17**	**23**	**27**	**36**	**38**	**46**
Duplex (Area)	220896	32068	25061	22321	21225	15770	14975	12751
Triplex (Area)	0	961	5289	6824	7865	8988	9342	10674

TFO concentration (nM)	0	10	50	60	70	80	90	100
Triplex (%)	**0**	**4**	**18**	**24**	**27**	**36**	**38**	**45**
Duplex (Area)	221412	33857	28501	23553	25153	19099	18565	16193
Triplex (Area)	0	1284	6166	7600	9465	10668	11363	13270

<u>Quantitative analysis of 5992-TFO</u>

20 mM $MgCl_2$

TFO concentration (nM)	0	10	50	60	70	80	90	100
Triplex (%)	**0**	**4**	**38**	**47**	**54**	**74**	**84**	**93**
Duplex (Area)	221428	26175	15255	10738	10779	4844	2854	1173
Triplex (Area)	0	962	9189	9630	12631	13594	15258	15979

TFO concentration (nM)	0	10	50	60	70	80	90	100
Triplex (%)	**0**	**5**	**35**	**48**	**55**	**78**	**88**	**95**
Duplex (Area)	221416	29407	16289	11179	10423	3962	2184	834
Triplex (Area)	0	1515	8663	10144	12647	13671	15679	17002

TFO concentration (nM)	0	10	50	60	70	80	90	100
Triplex (%)	**0**	**3**	**35**	**48**	**56**	**70**	**85**	**95**
Duplex (Area)	221325	32401	21340	14680	11668	6212	3315	1086
Triplex (Area)	0	1092	11572	13306	14705	14773	18506	19397

5 mM $MgCl_2$

TFO concentration (nM)	0	10	50	60	70	80	90	100
Triplex (%)	**0**	**4**	**35**	**49**	**65**	**79**	**94**	**97**
Duplex (Area)	221253	29002	17450	11924	7672	4386	1248	539
Triplex (Area)	0	1353	9546	11641	14477	16612	20079	19228

TFO concentration (nM)	0	10	50	60	70	80	90	100
Triplex (%)	**0**	**5**	**35**	**48**	**65**	**76**	**93**	**98**
Duplex (Area)	32140	28903	19913	13324	8374	6157	1518	386
Triplex (Area)	0	1436	10532	12542	15838	19745	21270	20428

TFO concentration (nM)	0	10	50	60	70	80	90	100
Triplex (%)	**0**	**4**	**39**	**49**	**65**	**78**	**95**	**95**
Duplex (Area)	26998	34974	18937	14765	9730	6555	1300	1242
Triplex (Area)	0	1630	12333	14458	17718	22681	24459	25155

Quantitative analysis of 5992-Ps1

20 mM $MgCl_2$

TFO concentration (nM)	0	10	50	60	70	80	90	100
Triplex (%)	**0**	**3**	**26**	**42**	**53**	**67**	**81**	**86**
Duplex (Area)	221434	29197	20021	13142	10651	6666	3165	2448
Triplex (Area)	0	922	6961	9482	12004	13626	13416	14451

TFO concentration (nM)	0	10	50	60	70	80	90	100
Triplex (%)	**0**	**3**	**27**	**47**	**60**	**75**	**78**	**84**
Duplex (Area)	221430	33502	23153	15407	10551	5946	5537	3660

Triplex (Area)	0	1045	8517	13596	15912	17383	19752	19791

TFO concentration (nM)	0	10	50	60	70	80	90	100
Triplex (%)	**0**	**3**	**29**	**46**	**60**	**77**	**82**	**83**
Duplex (Area)	221426	33189	23542	16277	10614	4769	4062	4142
Triplex (Area)	0	1058	9622	13966	15631	15973	18192	19724

---

5 mM $MgCl_2$

TFO concentration (nM)	0	10	50	60	70	80	90	100
Triplex (%)	**0**	**5**	**30**	**40**	**51**	**70**	**79**	**77**
Duplex (Area)	34910	30977	20393	15938	12920	6220	4116	4898
Triplex (Area)	0	1467	8610	10773	13265	14637	15865	16274

TFO concentration (nM)	0	10	50	60	70	80	90	100
Triplex (%)	**0**	**5**	**29**	**40**	**50**	**69**	**83**	**78**
Duplex (Area)	30839	27140	19717	15543	11753	5946	2849	4367
Triplex (Area)	0	1335	8174	10153	11981	13240	14061	15074

TFO concentration (nM)	0	10	50	60	70	80	90	100
Triplex (%)	**0**	**5**	**32**	**43**	**54**	**73**	**83**	**82**
Duplex (Area)	37332	29703	17669	16259	12223	6041	3815	3844
Triplex (Area)	0	1668	8388	12031	14377	16446	18173	17219

<u>Quantitative analysis of 5992-Ps2</u>

20 mM $MgCl_2$

TFO concentration (nM)	0	10	50	60	70	80	90	100
Triplex (%)	**0**	**5**	**40**	**52**	**57**	**84**	**92**	**93**
Duplex (Area)	31853	23625	16605	11268	10388	2671	1347	1208
Triplex (Area)	0	1344	10962	12325	14046	14193	16614	16778

TFO concentration (nM)	0	10	50	60	70	80	90	100
Triplex (%)	**0**	**6**	**37**	**51**	**61**	**84**	**92**	**92**
Duplex (Area)	34660	24478	16123	12815	9857	2872	1597	1551
Triplex (Area)	0	1436	9487	13317	15228	15268	19027	17229

TFO concentration (nM)	0	10	50	60	70	80	90	100

Triplex (%)	**0**	**6**	**37**	**51**	**60**	**80**	**91**	**89**
Duplex (Area)	34894	25096	16376	13770	10483	4012	1806	2302
Triplex (Area)	0	1629	9746	14080	15669	16340	17508	17887

---

5 mM $MgCl_2$

TFO concentration (nM)	0	10	50	60	70	80	90	100
Triplex (%)	**0**	**4**	**35**	**47**	**59**	**80**	**95**	**100**
Duplex (Area)	37448	32706	17455	13703	9438	4219	984	0
Triplex (Area)	0	1492	9461	12038	13726	16465	17743	18888

TFO concentration (nM)	0	10	50	60	70	80	90	100
Triplex (%)	**0**	**3**	**35**	**47**	**60**	**79**	**92**	**100**
Duplex (Area)	221375	32295	17541	14458	10049	4925	1621	0
Triplex (Area)	0	1133	9622	13008	15261	18088	19719	17316

TFO concentration (nM)	0	10	50	60	70	80	90	100
Triplex (%)	**0**	**4**	**38**	**49**	**62**	**79**	**91**	**100**
Duplex (Area)	221319	29577	16295	11990	8460	4227	1697	0
Triplex (Area)	0	1265	9945	11672	13831	16027	17446	17599

Table S2. The row datas of WST-8 assay (absorbance at 450 nm).

	Ps-scramble	205-TFO	205-Ps1	205-Ps2
1	0.794	0.556	0.355	0.380
2	0.709	0.551	0.363	0.388
3	0.880	0.550	0.360	0.395
4	0.888	0.528	0.350	0.363

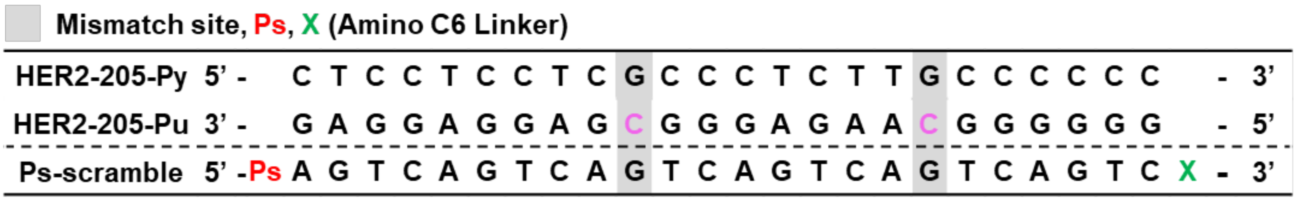

Figure S8. The sequence of Ps-scramble. The canonical design of psoralen-conjugated TFO (ref. 4, 5) was used as scramble TFO to show that the cell death response was induced in sequence selective manner.

Ref. 4

Y. Mikame, H. Eshima, H. Toyama, J. Nakao, M. Matsuo, T. Yamamoto, Y. Hari, J. A. Komano, A. Yamayoshi, *ChemMedChem*. **2023**, *18*, e202300348.

Ref. 5

K. M. Vasquez, J. M. Dagle, D. L. Weeks, P. M. Glazer, *J. Biol. Chem.* **2001**, *276*, 38536-38541
